# Normative distribution of corneal epithelial thickness on 9-mm OCT maps

**DOI:** 10.3389/fmed.2025.1572326

**Published:** 2025-07-14

**Authors:** Claudia R. Morgado, Marcony R. Santhiago

**Affiliations:** Department of Ophthalmology at University of Sao Paulo, São Paulo, Brazil

**Keywords:** corneal epithelium, optical coherence tomography, normal epithelium, cornea, normal eye

## Abstract

This prospective observational study comprehensively characterized the normative distribution of corneal epithelial thickness using 9-mm spectral-domain optical coherence tomography (SD-OCT) maps. Data were collected from 283 eyes of refractive surgery candidates who underwent a complete ophthalmologic evaluation, including SD-OCT imaging. The analysis revealed a mean central corneal epithelial thickness of 53.34 μm (SD ± 3.26 μm), exhibiting a heterogeneous distribution with significantly thicker measurements in inferior compared to superior regions. This distribution followed a Gaussian pattern, with the thickest measurements consistently observed in the central and inferior regions. The study also revealed statistically significant differences in central corneal epithelial thickness between males (54.11 μm) and females (52.64 μm). A weak but significant negative correlation was observed between superior epithelial thickness and age. This detailed analysis of corneal epithelial thickness across multiple zones within a large sample provides valuable normative data essential for clinical practice and future research in corneal pathology. The established normative reference values are critical for accurately identifying deviations in epithelial thickness associated with conditions such as keratoconus.

## Introduction

The corneal epithelium’s crucial metabolic functions and remarkable capacity for dynamic remodeling have long been acknowledged (1). Recent advances in imaging modalities, encompassing confocal microscopy, very high-frequency digital ultrasound and optical coherence tomography (OCT), have substantially enhanced our comprehension of epithelial thickness and its responsiveness to diverse pathological states (2, 3). Emerging evidence from histological analyses indicates that early pathological alterations in prevalent conditions such as keratoconus manifest within the epithelial layer (4, 5).

The widespread adoption of OCT has facilitated the rapid and efficient acquisition of reproducible, high-resolution 9 mm epithelial thickness maps (6). Prior research has established the clinical utility of epithelial mapping in various contexts, including optimizing refractive surgery protocols, predicting post-operative tissue remodeling, and improving the differentiation between normal and ectatic corneas, particularly in the early stages of keratoconus (7, 8). The corneal epithelium demonstrates an adaptive remodeling capacity, dynamically responding to alterations in anterior corneal curvature or underlying stromal abnormalities. Distinct epithelial thickness patterns have been described, aiding in the discrimination between ectatic and non-ectatic corneal conditions (9). However, despite its increasing clinical relevance, a comprehensive understanding of normative epithelial thickness distribution across a large, randomly selected population remains elusive.

This study aims to comprehensively define the normative distribution of corneal epithelial thickness within 9 mm OCT maps. By characterizing the mean values and 2.5th and 97.5th percentile limits through the analysis of a substantial sample size, we aim to provide a thorough understanding of normative epithelial thickness distribution across a large, randomly selected population.

## Materials and methods

### Study design and subjects

This is a prospective comparative observational study approved by the institutions’ ethics committee (University of Sao Paulo) and by the Brazilian National ethics and research committee (IRB number 6.003.562). This study also followed the tenets of the Declaration of Helsinki and was carried out in compliance with institutional ethics guidelines for obtaining patient samples after prior informed written consent. Informed consent was obtained from all patients.

### Patient selection and imaging

Patients were randomly included if they presented as refractive surgery candidates and had undergone a comprehensive ophthalmic examination at the time of their clinical evaluation, including a slit lamp examination, dilated fundus examination, and determination of their manifest and cycloplegic refractions, as well as imaging with an anterior segment optical coherence (Avanti Optovue). This patients comprised 283 eyes of 283 unique participants—147 female eyes and 136 male eyes. The mean age was 34.72 ± 9.69 years for women (range, 16–67 years) and 34.97 ± 10.65 years for men (range, 14–68 years). Table 1 shows this age distribution. All examinations on every device were performed on the same day for each participant by the same experienced examiner (M.R.S.) between January 2021 and October 2024 in a single private practice setting.

**TABLE 1:** Age and gender distribution of participants.

Age group (years)	Female (*n*)	Male (*n*)	Total (*n*)
< 20	5	4	9
20–29	45	40	85
30–39	55	50	105
40–49	30	30	60
50–59	10	10	20
≥ 60	2	2	4
Total	147	136	283

To ensure independence of data and reduce correlation between eyes, this study exclusively included one eye per participant, randomly selecting the right eye for analysis. Patients were excluded if they had imaging of inadequate quality, missing specific data used for analysis, had a history of previous ocular surgery, or had any ophthalmic abnormality that would alter surgical decision-making regardless of their risk of corneal ectasia (e.g., corneal scars) or pregnancy. Contact lens wear was discontinued at least 7 days for soft contact lens and 15 days for hard contact lens before initial screening. This was applied to avoid transient epithelial and corneal changes induced by lens wear, which could bias the measurements and compromise the accuracy of normative thickness assessment.

All patients underwent a complete ophthalmological examination, including uncorrected distance visual acuity (UCVA), best spectacle-corrected visual acuity (BSCVA), and manifest refraction. An experienced examiner (CRM) carefully performed the refraction.

A spectral domain SD-OCT system (Avanti model; OptoVue, Inc.) with a corneal adaptor lens was used to acquire each of the epithelial variables used in this study. This device has a working wavelength of 840 nm and operates at a scan speed of 70,000 axial scans per second. Equipped with an add-on lens, this system makes corneal measurements with the “PachymetryWide” scan mode, consisting of B-scans evenly in eight radial directions at a length of 9 mm centered at the pupil center. The device was used according to the user’s manual, measurements were performed under standard ambient photopic conditions, consistent with clinical room lighting. The scans were triggered manually after the alignment procedure was completed. Participants were asked to sit back to ensure measurement autonomy, and the scan unit was thoroughly reset before each subsequent scan. The data were valid if the measurement outcomes showed sufficient image signals and good quality, (as per Optovue software quality indicators), full corneal coverage, and absence of motion or decentration artifacts.

The mean epithelial values in 25 zones of the inferior central, temporal, and nasal zones and the superior central temporal and nasal were identified with a high degree of precision. The values of 2 to 5 mm, 5 to 7 mm, and 7 to 9 mm were meticulously determined for each measurement, ensuring the thoroughness of our research process.

**FIGURE 1:**
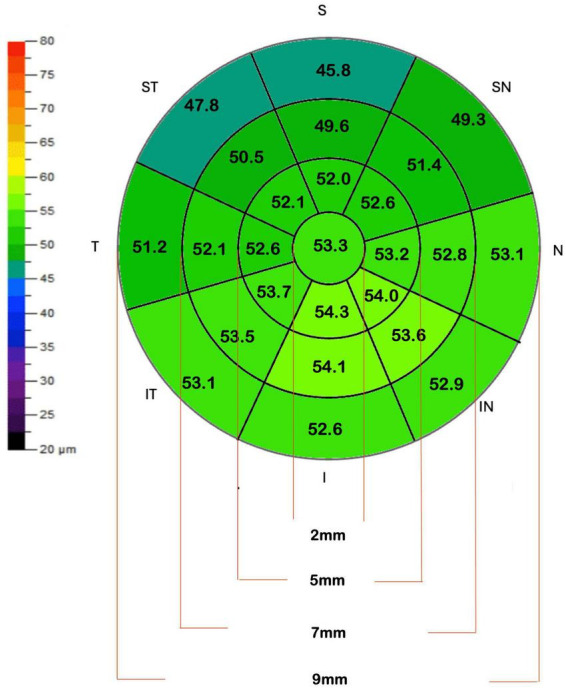
Epithelial thickness map representing the average measurements of the study population. The colors indicate the variation in epithelial thickness according to the presented scale. The central, paracentral, mid-periphery and peripheral zones have diameters of 2, 5, 7, and 9 mm, respectively.

Below the specific areas of the epithelial map analyzed in this study:

1. Central epithelial thickness of the map (2 mm circular zone);
2. Inferior paracentral (2–5 mm);
3. Inferior mid-periphery (5–7 mm)
4. Inferior periphery (7–9 mm);
5. Inferior nasal paracentral (2–5 mm);
6. Inferior nasal mid-periphery nasal (5–7 mm);
7. Inferior nasal periphery (7–9 mm)
8. Inferior temporal paracentral (2–5 mm);
9. Inferior temporal mid-periphery nasal (5–7 mm);
10. Inferior temporal periphery (7–9 mm)
11. Superior paracentral (2–5 mm);
12. Superior mid-periphery (5–7 mm)
13. Superior periphery (7–9 mm);
14. Superior nasal paracentral (2–5 mm);
15. Superior nasal mid-periphery nasal (5–7 mm);
16. Superior nasal periphery (7–9 mm)
17. Superior temporal paracentral (2–5 mm);
18. Superior temporal mid-periphery nasal (5–7 mm);
19. Superior temporal periphery (7–9 mm)
20. Nasal paracentral (2–5 mm);
21. Nasal mid-periphery (5–7 mm)
22. Nasal periphery (7–9 mm);
23. Nasal paracentral (2–5 mm);
24. Nasal mid-periphery (5–7 mm)
25. Nasal periphery (7–9 mm);

The mean of each of these measurements was analyzed and a mean map of the population was reconstructed on a color scale (Figure 1).

In addition, we carefully accounted for the mean of the measurements of other variables generated in the device.

26. Minimum (thinnest) epithelial thickness of the map
27. Maximum (thickest) epithelial thickness of the map
28. Difference between the minimum and maximum epithelial thickness of the map
29. Inferior epithelial thickness (average of lower measurements within 2–7 mm)
30. Superior epithelial thickness (average of upper measurements within 2–7 mm)
31. Difference between the inferior and superior measurements
32. Standard deviation of the epithelial thickness of the map

In total, therefore, 32 epithelial variables were measured and analyzed in all eyes included in the study.

Descriptive statistics were employed to analyze the means, standard deviations, and the 2.5th and 97.5th percentile limits for all eyes. Categorical variables were summarized as counts and percentages (%), while continuous variables were reported as means and standard deviations. To assess differences between groups, a paired sample *t*-test was conducted. The Pearson correlation coefficient was used to evaluate the correlation between the thinnest and thickest epithelial points with spherical equivalent, age, and white-to-white measurements. Normality of the data was assessed using the Shapiro–Wilk test and the Kolmogorov–Smirnov test to check for statistical collinearity. A significance level of *p* < 0.01 was set for all statistical tests. The data were analyzed using the Statista software program.

## Results

**FIGURE 2:**
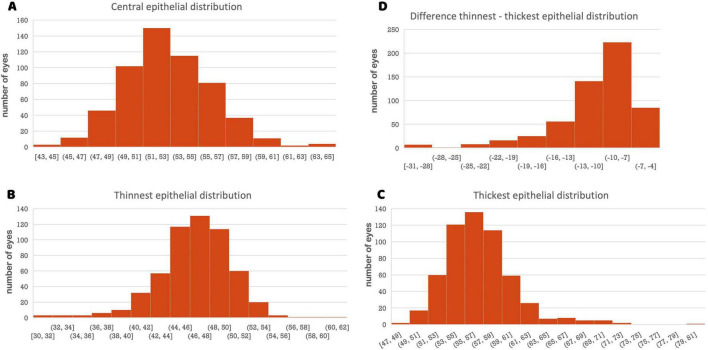
**(A)** Histogram of the central epithelial thickness distribution. The *X*-axis represents epithelial thickness (μm), and the *Y*-axis represents the number of eyes. **(B)** Histogram of the thinnest epithelial distribution. **(C)** Histogram of the thickest epithelial distribution. **(D)** Histogram of the difference thinnest–thickest epithelial distribution.

This cross-sectional study included 283 eyes from 283 individual participants. The mean keratometric values were 43.01 ± 1.64 D for K1 and 44.34 ± 1.74 D for K2. The mean spherical equivalent was −2.74 ± 2.82 D (range −8.00 to +4.00). Table 2 presents the mean epithelial thickness, standard deviation, and 2.5th and 97.5th percentile values for all measured zones. Analysis revealed a mean central corneal epithelial thickness of 53.34 μm (SD ± 3.26 μm), with inferior measurements averaging 3.49 μm thicker than superior measurements (Figure 1). Epithelial thickness exhibited heterogeneous distribution, with the thickest measurements consistently observed in central and inferior regions. This inferior thickening trend was further substantiated by analysis of the annular zones (2–5 mm, 5–7 mm, and 7–9 mm), which consistently showed thicker measurements in central areas across all inferior, inferotemporal, inferonasal, temporal, nasal, superior, superonasal, and superotemporal regions. A nasal thickening trend was also observed horizontally across all zones. The thickest epithelial regions were identified as the inferonasal and inferior paracentral (2–5 mm) and mid-peripheral (5–7 mm) zones (Figure 1). The distribution of mean central epithelial thickness, as well as the thinnest and thickest epithelial thicknesses, followed a Gaussian distribution. Histograms confirmed a normal distribution of the measurements, demonstrating symmetry and central tendency around the mean, indicative of adherence to a classic statistical pattern (Figure 2).

**TABLE 2:** Mean epithelial data in all eyes included in the study 283.

Epithelial data (μm)	Mean ± standard deviation	Range	Percentile 2.5	Percentile 97.5
Epithelial central	53.34 ± 3.26	43–64	47	60
Epithelial minimum	46.84 ± 3.95	30–57	35	53
Epithelial maximum	57.18 ± 3.97	47–81	51	67
Epithelial min–max	−10.34 ± 4.65	−30 to −4	−23	−5
Epithelial std dev	2.15 ± 0.94	0.8–6.6	1	4.7
Epithelial inferior	54.23 ± 3.20	45–66	49	61
Epithelial superior	50.74 ± 3.05	42–66	46	56

Table 3 shows comparison between female and male epithelial data. The epithelial measurement values of men are consistently thicker than those of women, although only the central measurement has reached a statistically significant difference. The difference in central epithelial thickness between sexes was statistically significant (*p* = 0.00013), with males showing a higher mean (54.11 μm, 95% CI: 53.60–54.62) compared to females (52.64 μm, 95% CI: 52.10–53.18). The effect size was moderate (Cohen’s *d* = 0.46), supporting the clinical relevance of this difference.

**TABLE 3:** Female vs. male epithelial data.

Epithelial data (μm)	Female 147 eyes Mean ± standard deviation	Male 136 eyes Mean ± standard deviation	*p*-value
Epithelial central	52.64 ± 3.30	54.11 ± 3.03	0.00013*
Epithelial minimum	46.59 ± 3.71	47.10 ± 4.19	0.284
Epithelial maximum	56.82 ± 3.72	57.58 ± 4.21	0.108
Epithelial min-max	−10.22 ± 4.48	−10.48 ± 4.84	0.643
Epithelial std dev	2.11 ± 0.91	2.19 ± 0.98	0.469
Epithelial inferior	53.95 ± 3.12	54.54 ± 3.20	0.128
Epithelial superior	50.34 ± 2.73	51.18 ± 3.32	0.020

*Statistically significant *p* < 0.007.

**FIGURE 3:**
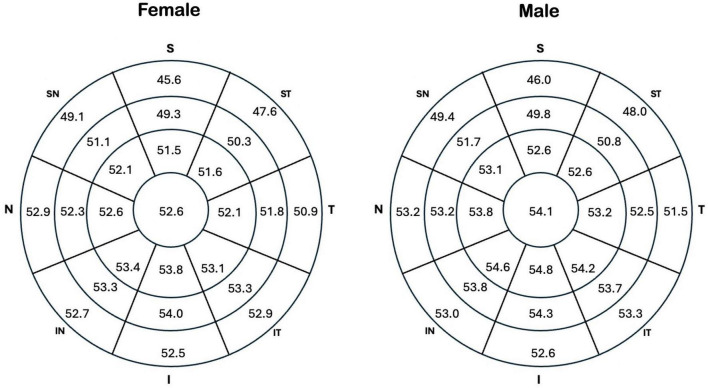
Epithelial thickness maps representing the average measurements of the study population, stratified by sex.

Comparison of epithelial thickness measurements between 136 male eyes and 147 female eyes participants revealed a statistically significant difference (*p* = 0.00013) in central corneal epithelial thickness, with males exhibiting a mean of 54.11 μm (SD ± 3.03 μm) compared to 52.64 μm (SD ± 3.30 μm) in females. No statistically significant sex-based differences were observed for minimum (*p* = 0.284) and maximum epithelial thickness (*p* = 0.108), the difference between minimum and maximum epithelial thickness (*p* = 0.643), or the standard deviation of epithelial thickness measurements (*p* = 0.469). A marginally significant difference (*p* = 0.020) was observed in superior epithelial thickness, with males showing a mean of 51.18 μm (SD ± 3.32 μm) compared to 50.34 μm (SD ± 2.73 μm) in females. No significant difference in inferior epithelial thickness was found between the sexes (*p* = 0.128). Furthermore, Figure 3 highlights that the 25 zones analyzed in the epithelial map are consistently thicker in men than in women (*p* < 0.007 for all comparisons). The *p*-value threshold of 0.007 corresponds to a Bonferroni correction for the seven primary comparisons between sexes (central, minimum, maximum, min-max, SD, inferior, and superior).

**FIGURE 4:**
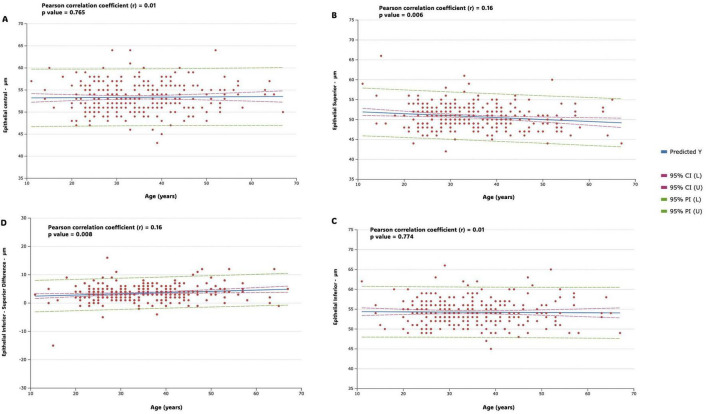
Figure shows correlation strength and multiple linear regression for epithelial variables in the study population. Predicted values (predicted Y, blue line), 95% confidence intervals (horizontal red dotted line), and 95% prediction intervals (horizontal green dotted line) are represented. Regression lines, along with confidence and prediction intervals, are visually represented in the figures. **(A)** Central epithelial thickness **(B)** inferior-superior epithelial difference **(C)** superior epithelial thickness **(D)** inferior epithelial thickness.

Figure 4 shows correlation strength and multiple linear regression for epithelial variables (Central epithelial thickness, Inferior-superior epithelial difference, superior and inferior epithelial thickness) in the study population. In the analysis of the correlation of the central and inferior epithelial measurement with age, the results revealed a negligible correlation in both cases (*r* = 0.01, *p* = 0.765 and *r* = 0.01, *p* = 0.774, respectively); while the results revealed a weak, but significant, negative correlation of the superior epithelial measurement with age (*r* = −0.16, *p* = 0.006). In the analysis of the correlation of the inferior-superior central epithelial differences with age, the results revealed a weak, but also significant, positive correlation (*r* = 0.16, *p* = 0.008).

**FIGURE 5:**
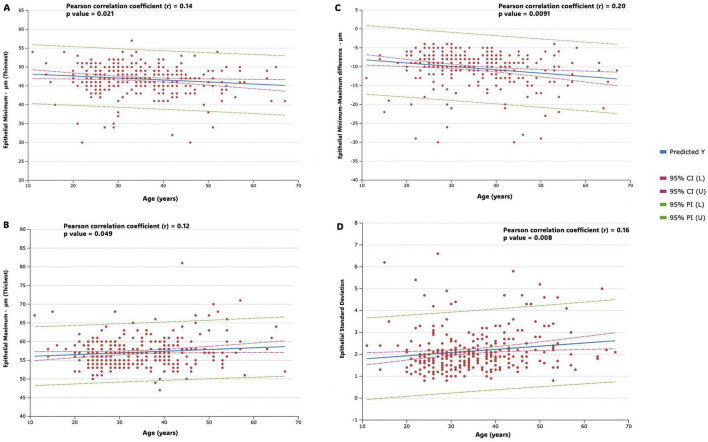
Figure shows correlation strength and multiple linear regression for epithelial variables in the study population. Predicted values (predicted Y, blue line), 95% confidence intervals (horizontal red dotted line), and 95% prediction intervals (horizontal green dotted line) are represented. Regression lines, along with confidence and prediction intervals, are visually represented in the figures. **(A)** Epithelial minimum (thinnest) **(B)** epithelial maximum (thickest) **(C)** epithelial minimum- maximum difference **(D)** epithelial standard deviation.

Figure 5 shows correlation strength and multiple linear regression for epithelial variables (epithelial Minimum, epithelial Maximum, Epithelial Minimum- Maximum difference and epithelial standard deviation) in the study population. In the analysis of the correlation of the minimum (thinnest) and maximum (thickest) measurements with age, the results revealed a weak and not significant correlation in both cases (*r* = −0.14, *p* = 0.021 and *r* = 0.12, *p* = 0.049, respectively); while the results revealed a weak, but significant, negative correlation of the minimum-maximum difference measurement (*r* = −0.20, *p* = 0.009) and a weak, but significant, positive correlation of standard deviation measurement (*r* = 0.16, *p* = 0.008) with age (Figure 5).

## Discussion

The identification of this Gaussian distribution for the mean epithelial thickness, as well as for the thinnest and thickest points, presents several important implications for clinical practice and research. Firstly, this distribution facilitates the establishment of normative reference values that can be widely utilized in clinical assessments, enabling ophthalmologists to accurately identify both normal and abnormal variations in epithelial thickness. This is particularly crucial for early diagnostic efforts, where deviations from normative values may signal the onset of pathological conditions, such as keratoconus. Additionally, the normality of the data justifies the use of traditional statistical methods, which assume a normal distribution, thereby enhancing the robustness of analyses conducted through tests like *t*-tests and ANOVA. In terms of ocular health, the evidence that epithelial thickness exhibits a Gaussian distribution in a healthy population indicates a stable barrier function and suggests a state of physiological homeostasis, in which the epithelial layers are adequately adapted to environmental conditions and physiological demands. This stability reflects the epithelium’s compensatory ability to mask stromal irregularities and underscores the clinical significance of established reference ranges, as deviations from normal thickness patterns may signal early signs of keratoconus and other ectatic disorders, thereby serving as valuable indicators in the assessment of corneal integrity.

Recognition of normative distribution patterns not only enhances diagnostic precision but also informs future research directions in ophthalmology, allowing for a deeper exploration of correlations between demographic factors and ocular variables. Clinicians evaluating suspect cases can utilize these data to identify significant deviations from typical epithelial thickness patterns. Measurements falling below the 2.5th percentile or above the 97.5th percentile for minimum thickness, maximum thickness, or standard deviation warrant further investigation. Our findings demonstrate the clinical utility of establishing normal ranges for parameters like epithelial Min–Max in monitoring keratoconus progression. The 2.5th and 97.5th percentiles we identified (−23 and −5, respectively) corroborate Santhiago et al.’s (10) findings, suggesting that values at or below the 2.5th percentile may signal impending progression. Early identification of patients with values outside these ranges allows for more timely interventions and better management of keratoconus progression.

This study yielded a mean central corneal epithelial thickness of 53.34 ± 3.26 μm. Comparison with Ma et al. (11), who utilized the RTVue-XR system, reveals a mean central epithelial thickness of 54.5 ± 5.9 μm in their normal-eyed cohort–a value slightly exceeding the present study’s findings. This difference likely reflects inherent inter-study variability. Focusing on the virgin eyes from Feng et al. (12), who employed both Avanti and Anterion OCT systems, the Avanti device yielded a mean central corneal epithelial thickness of 55.60 ± 3.26 μm. This value is notably higher than both the current studies and Ma et al.’s (11) findings. Conversely, using the Anterion in the same virgin-eye group, Feng et al. (12) measured a mean central epithelial thickness of 51.59 ± 3.27 μm. This discrepancy highlights the substantial influence of device technology on measured central corneal epithelial thickness. The AlTurki et al. (13) study, utilizing the MS-39 AS-OCT system, reported mean central epithelial thicknesses exhibiting variations based on age and sex, generally concurring with the observation of greater thicknesses in males and older participants. They found a mean epithelial value for the 3 mm central area was 52.1 ± 4.08 μm across the groups. Using VHFU technology, Reinstein et al. (14) showed that the average central CET was 53.4 ± 4.6 μm, comparable to SD-OCT used in this study while the work conducted by Tañá-Rivero et al. (15), using the Cirrus 5000 HD-OCT, showed a much thinner average, 48.16 ± 3.25 μm in the central epithelial thickness for eyes considered suitable for refractive surgery.

While direct numerical comparisons are constrained by methodological variations, analyzed regions, and patient population characteristics across these studies, the collective data emphasize the significant variability in central corneal epithelial thickness, influenced by factors such as device technology, age, sex, and underlying corneal conditions. The larger sample size in the present study affords more robust normative data, highlighting the imperative for standardized methodologies in future studies to enhance inter-study comparability.

Prior research has consistently demonstrated the reliability and reproducibility of corneal epithelial thickness measurements using spectral-domain optical coherence tomography (SD-OCT), aligning with the methodology employed in this study (11, 16, 17). Furthermore, a consensus exists across studies, utilizing diverse populations and technologies, regarding the thicker epithelial thickness observed inferiorly and nasally.

Several hypotheses address the observation of thicker inferior corneal epithelium. While tear film pooling might artificially inflate inferior measurements, (18) studies using very high-frequency digital ultrasound techniques that exclude this effect (2, 14) still demonstrate inferior thickening, indicating other contributing factors. One such factor is the sustained mechanical stress from the upper eyelid on the superior cornea, as proposed by Du et al. (19). Their hypothesis suggests that the upper eyelid’s greater coverage and gravitational influence induce chronic pressure, leading to superior thinning. Conversely, the reduced pressure inferiorly might allow for relatively thicker epithelium.

Interestingly, while central and inferior epithelial thickness remain largely unchanged with age, the superior epithelium exhibits a significant, albeit weak, negative correlation with age, demonstrating thinning over time. Our study presented the larger sample size with this OCT model to rigorously demonstrate this age-related superior epithelial thinning, even with the methodological constraint of using only one eye per patient. This finding further supports the hypothesis that the combined effects of upper eyelid weight and the lifetime of blinking contribute to a physiologically thicker inferior and thinner superior corneal epithelium. The increased variability in epithelial measures, such as the min-max difference and standard deviation, observed in older individuals, likely reflects the higher cellular turnover and tear film dynamics in aging eyes.

Women consistently exhibit thinner corneas across all epithelial map areas (Figure 3). This finding aligns with previous research documenting thicker epithelial measurements in males compared to females, with differences reaching up to 2.2 μm (6, 13, 20). Our study corroborates this trend, showing a statistically significant 1.45 μm difference in central epithelial thickness between sexes. This sexual dimorphism is likely influenced by hormonal factors, as evidenced by Kelly et al. (21), who demonstrate the profound and multifaceted effects of female sex hormones on corneal structure and function throughout a woman’s lifespan. The variations in hormone levels across the menstrual cycle, pregnancy, and menopause significantly impact corneal thickness, intraocular pressure, and tear film dynamics. Therefore, the thinner corneal epithelium observed in women in our study likely reflects the influence of these hormonal variations.

This study utilized 9-mm diameter corneal mapping, offering a more comprehensive assessment of corneal thickness compared to the 6-mm maps common in previous studies. Expanding the mapping area to 9 mm is particularly advantageous for evaluating corneal conditions like keratoconus and post-refractive surgery where peripheral changes are clinically significant. Latifi and Mohammadi (17) using the same RTVue-XR system with both 6-mm and 9-mm algorithms, confirmed good repeatability for both central and peripheral measurements. This wider mapping approach, as also employed by Hashmani et al. (6) overcomes limitations inherent to smaller diameter scans, enhancing the detection of peripheral corneal changes. However, both Latifi and Mohammadi (17) and Hashmani et al. (6) observed challenges related to measurement consistency in peripheral zones, potentially due to factors such as tear film interference and signal acquisition. Therefore, standardized protocols and analysis methods are crucial to maximize the benefits of 9-mm mapping while mitigating peripheral measurement errors and improving clinical interpretation. The theoretical advantage of 9-mm mapping lies in its improved assessment of peripheral corneal changes; however, rigorous standardization of techniques is essential for reliable clinical application.

While this study’s design includes potential limitations such as selection bias from recruitment within a single refractive surgery practice, these concerns are mitigated by several factors. The large sample size enhances the generalizability of the findings. Furthermore, the strict inclusion criteria—requiring eyes with good surface quality and high-quality examinations—reduces the influence of confounding factors such as poor image quality or surface irregularities that could affect thickness measurements. To avoid the potential confounding effect of correlated data from paired eyes within the same individual, only one eye (randomly selected) was included per participant. This approach ensured statistical independence among data points, enhancing the robustness of the analysis.

Additionally, the age distribution in our cohort is skewed toward younger adults, reflecting the typical demographic of refractive surgery candidates. Although this does not fully represent the general population, it provides a relevant normative reference for healthy, treatment-naïve eyes within this age range. These factors limit the external validity of our findings but were necessary to ensure internal consistency. Future population-based studies including a broader age range and refractive error spectrum are warranted to further validate these results. The cross-sectional design limits the ability to draw causal inferences regarding age-related changes; however, the robust sample size strengthens our descriptive conclusions about these relationships.

Finally, this study provides a comprehensive characterization of normative corneal epithelial thickness distribution using 9-mm SD-OCT maps in a substantial sample size. The observed Gaussian distribution for mean, minimum, and maximum thickness establishes robust reference ranges for clinical assessment and aids in early detection of pathological conditions. The identified differences in epithelial thickness based on sex and the minor age-related trends highlight the need to consider these factors in clinical evaluations. The normative values presented will enhance diagnostic accuracy and contribute to advancing our understanding of corneal pathology. Future research with standardized methodologies involving diverse ethnic populations backgrounds is warranted to determine whether the normative epithelial thickness values identified here are consistent across different demographic groups.

## Data Availability

The original contributions presented in this study are included in this article/supplementary material, further inquiries can be directed to the corresponding author.
